# Prognostic significance of preoperative albumin to fibrinogen ratio associated nomograms in patients with breast invasive ductal carcinoma

**DOI:** 10.1097/MD.0000000000020681

**Published:** 2020-06-26

**Authors:** Lihua Zheng, Yaheng Zhao, Feng Liu, Peng Liu, Wei Li, Yan Yang, Hongsong Zhang, Yunjiang Liu

**Affiliations:** a Department of Breast Surgery, the Fourth Hospital of Hebei Medical University; b Department of General Surgery, Hebei Key Laboratory of Colorectal Cancer Precision Diagnosis and Treatment; c Department of Vascular Surgery; d Department of General Surgery, the First Hospital of Hebei Medical University, Hebei, Shijiazhuang, China.

**Keywords:** albumin to fibrinogen ratio, breast invasive ductal carcinoma, prognostic nomogram

## Abstract

Plasma albumin to fibrinogen ratio is involved in human cancer, but its prognostic significance in breast cancer is controversy. In the context of breast invasive ductal carcinoma, this research aims to retrospectively evaluate by preoperative plasma albumin to fibrinogen ratio (AFR) and forecast oncological outcome and recurrence.

This retrospective study comprised 230 patients with non-metastatic breast invasive ductal carcinoma who underwent surgery between January 2009 and April 2012 in Fourth Hospital of Hebei Medical University. Patients were categorized base on an optimal value of preoperative plasma fibrinogen (Fib) and albumin. Progression-free and cancer-specific survival were assessed using Kaplan–Meier method. The associations between albumin to fibrinogen ratio and clinical outcomes were assessed with univariate and multivariate analysis. A number of risk factors were used to form nomograms to evaluate survival, and Harrell concordance index (C-index) was used to evaluate the predictive accuracy.

Plasma AFR was significantly associated with diminished disease-free survival (DFS) and overall survival (OS). Multivariate analysis revealed that plasma AFR was an independent prognostic indicator for DFS (HR = 1.346; 95% CI: 1.107–1.636; *P* = .03) and overall survival (OS) (HR = 1.485; 95% CI: 1.106–1.993; *P* = .008). Two prediction model of 3-, 5-years OS and DFS based on the AFR was developed.

Elevated preoperative plasma AFR is an independent prognostic factor for oncological outcomes in patients with breast invasive ductal carcinoma. The formulated nomogram showed superior predictive accuracy for DFS and OS.

## 1 Introduction

Breast cancer is a complex disease which is found as the second cause of cancer-associated death among women and breast cancer has become a main health burden owing to the high rates of morbidity and cancer related mortality among women. Systemic inflammatory markers have also been reported to have a prognostic association with breast cancer. The systemic inflammatory markers investigated include single markers such as C-reactive protein,^[1]^ albumin,^[2]^ neutrophils,^[3]^ platelets,^[4]^ lymphocytes,^[5]^ and fibrinogen.^[6]^

Fibrinogen is a 340 kD glycoprotein consisted of 3 pairs distinct polypeptide chains, α, β, and γ. It is mainly synthesized in liver by hepatocytes and can be degraded by plasmin to form fibrinogen degradation products. Albumin is mainly produced by hepatocytes, however, extrahepatical synthesis by epithelial cells and tumor cells has been described.^[7]^ Fibrinogen (Fib) is an essential protein for coagulation cascade as well as an acute-phase reaction protein in response to systemic inflammation. Fibrinogen and albumin levels correlated with tumor stage and prognosis in several cancer types.^[8]^ Hence, preoperative albumin to fibrinogen ratio (AFR) level is a useful predictor for severe postoperative complications in elderly gastric cancer subjects after radical laparoscopic gastrectomy.^[9]^

Whether AFR can serve as a predictor for breast invasive ductal carcinoma patients still remains controversial. The purpose of this study is to further assess the predictive value of AFR in postoperative breast invasive ductal carcinoma and to formulate a new prognostic model through developing a nomogram. Then, according to the formulated nomogram, 2 novel nomogram-based disease-free survival (DFS) and overall survival (OS) was refined.

## 2 Methods

### 2.1 Patients

This retrospective study was approved by the Medical Institutional Ethics Committee of Hebei province and the Fourth Hospital of Hebei Medical University hospital (ethics code is 2019MEC111). The study included 230 women who underwent primary surgery at department of breast surgery in the Fourth Hospital of Hebei Medical University from January 2009 to April 2012, without any anticancer therapy before the operation, including radiation therapy, systemic chemotherapy, or hormone therapy.

### 2.2 Study design

All patients were followed up after surgery until the date of death or July 2016. The pathologic T: Tumor N: node M: metastasis (TNM) stage was judged by the 7th American Joint Committee on Cancer.^[10,11]^ The Ki-67 index was scored as high when 30% or more of the tumor cells were expressed. Analyses for estrogen receptor (ER), progesterone receptor (PR), and human epidermal growth factor receptor 2 (HER-2) were conducted according to the recommended guidelines of the American Society of Clinical Oncology and College of American Pathologists.^[12,13]^ Appropriate adjuvant treatments after the surgery were conducted according to the standard guidelines. Most of the hormone receptor (HR) positive patients received the adjuvant hormone therapy for 5 years at least. But none of the patients received the HER2-targeted adjuvant therapy.

### 2.3 Data collection

The following data were extracted and recorded from our database: clinicopathological features, including age, TNM, lymphovascular invasion nuclear grade, ER, PR, HER2, Ki-67, p53.

### 2.4 Laboratory tests

Fasting blood samples from each participant were before the operation. Blood cell analyses including white blood cell (WBC), red blood cell (RBC), hemoglobin (Hb), platelet (PLT), and albumin, Fib.

### 2.5 Statistical analysis

All statistical analyses and random allocation were performed by Empower Stats and R project version 3.3.3 (http://www.r-project.org/). The cutoff value of AFR was determined by median. The OS was compared by Kaplan–Meier curves and analyzed using the log-rank test via Empower Stats. The univariate and multivariate analyses and hazard ratios (HRs) were used by Cox proportional hazards regression model to find its independent prognostic risks, and *P* < .05 was considered as statistically significant difference. Two novel prognostic nomograms based on AFR for DFS and OS was formulated by Empower Stats. Its predictive performance was measured by concordance index (C-index), calibration curve, and decision curve analysis (DCA) was formulated by R project version.

## 3 Results

### 3.1 List the clinical characteristics of the patients and the clinicopathologic correlations

The median age of the 230 female patients was 48 years (range, 27–81). The median follow-up was 67 months (range, 7–81 months). Means, standard deviations, and ranges of laboratory results for albumin, fibrinogen, and AFR were 41.29 ± 3.39 g/d (range, 31.4–50.6 mg/dL), 3.21 ± 0.54 g/L (range, 1.97–4.93 g/L), and 13.24 ± 2.43 (range, 7.92–19.40), respectively. The optimal values for AFR were 13.1 by median (Table 1).

**Table 1:**
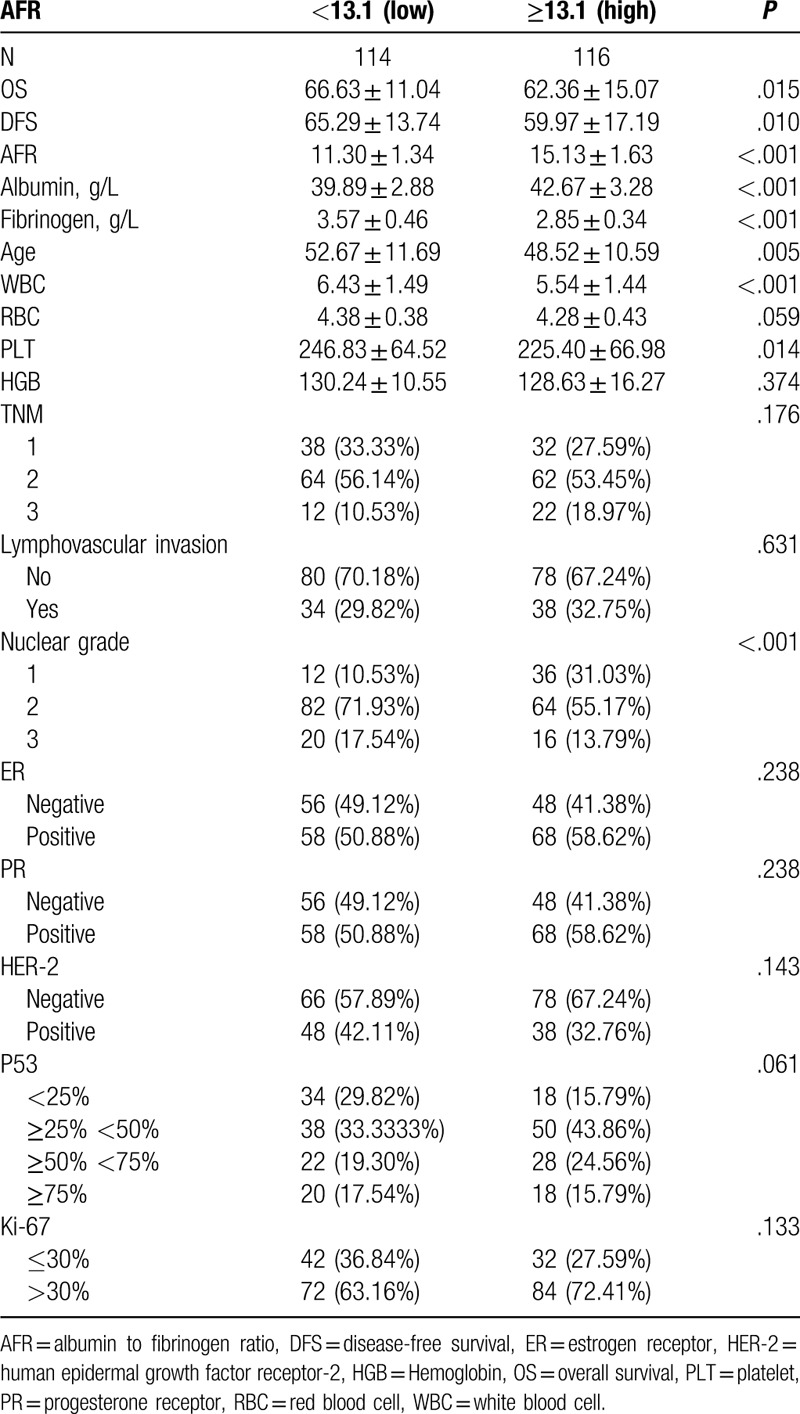
Clinical characteristics of patients.

### 3.2 Prognostic value of AFR

The higher levels of AFR showed significantly unfavorable DFS and OS. The clinicopathological parameters for the prediction of DFS and OS were further investigated by univariate analysis with the Cox regression model. In the univariate analysis, AFR was significantly associated with DFS and OS (Table 2). These significantly associated variables were used for the multivariate Cox regression model. In the DFS and OS models, AFR remained powerful and independent prognostic factors for patients with breast invasive ductal carcinoma (Table 3).

**Table 2:**
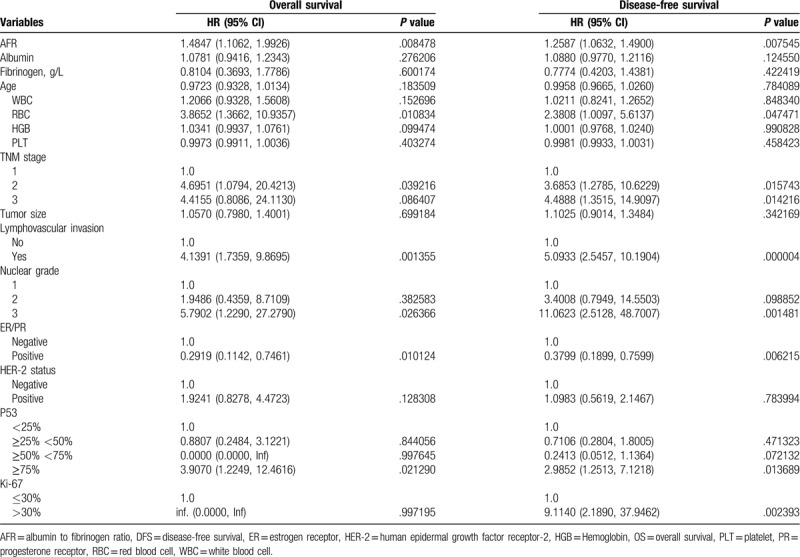
Univariate analysis of OS and DFS.

**Table 3:**
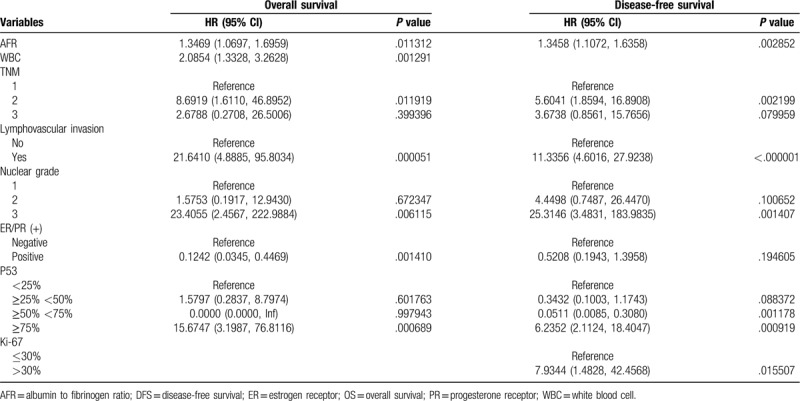
Multivariable Cox regression analysis of OS and DFS.

#### 3.2.1 Prognostic value of AFR by 2 classification

**Figure 1:**
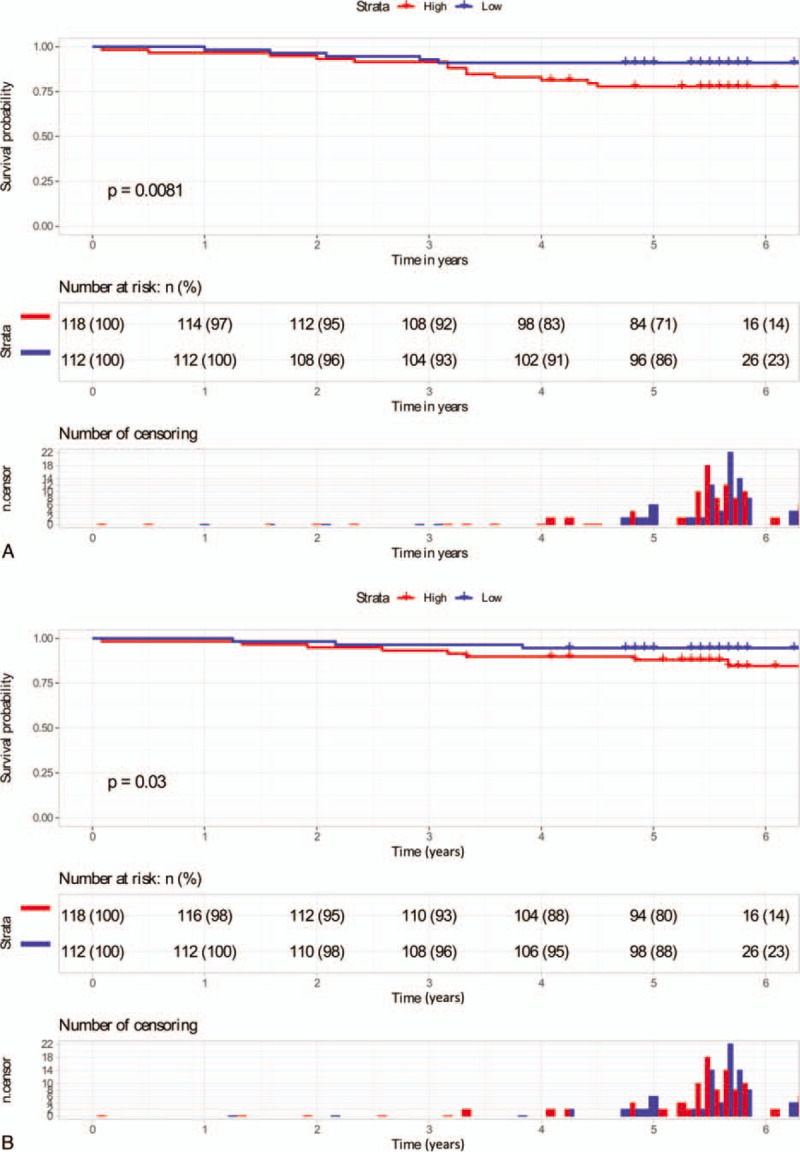
Kaplan–Meier survival analysis of DFS (A) and OS (B) according to low and high groups. DFS = disease-free survival, OS = overall survival.

To give a reasonable stratification of OS, the patients were divided into 2 groups on the basis of median value of AFR: a low-risk group (AR < 13.1, N = 114) and a high-risk group (AFR ≥ 13.1, N = 116). The median OS of the low-risk group and high-risk group is 66.63 months and 62.36 months, respectively. The median DFS of the low-risk group and high-risk group is 65.29 months and 59.97 months, respectively. The DFS and OS were significantly different among the 2 subgroups (*P* < .05). Patients with higher AFR had shorter OS (Fig. 1A) and DFS (Fig. 1B).

### 3.3 Novel prognostic nomogram for OS and DFS prediction

**Figure 2:**
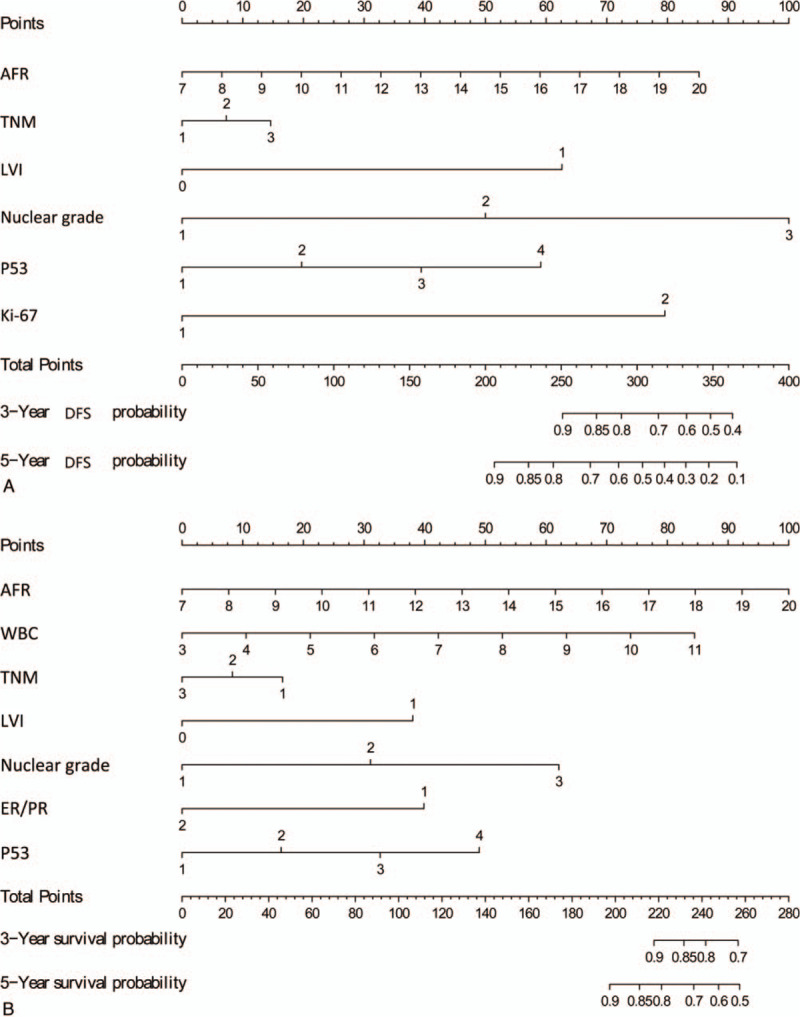
Nomogram model predicting 3- and 5-year DFS (A) and OS (B) in breast invasive ductal carcinoma patients. The nomogram was used summing the points identified on the points scale for each variable. The total points projected on the bottom scales indicate the probability of 3- and 5-year survival. ER/PR (+): 1 = negative, 2 = positive; P53 1 = <25%, 2 = ≥25%, <50%, 3 = ≥50%, <75%, 4 = ≥75%; Ki-67 1 = ≤30%, 2 = >30%, Lymphovascular invasion 0 = NO, 1 = Yes. DFS = disease-free survival, OS = overall survival; ER = estrogen receptor; PR = progesterone receptor.

**Figure 3:**
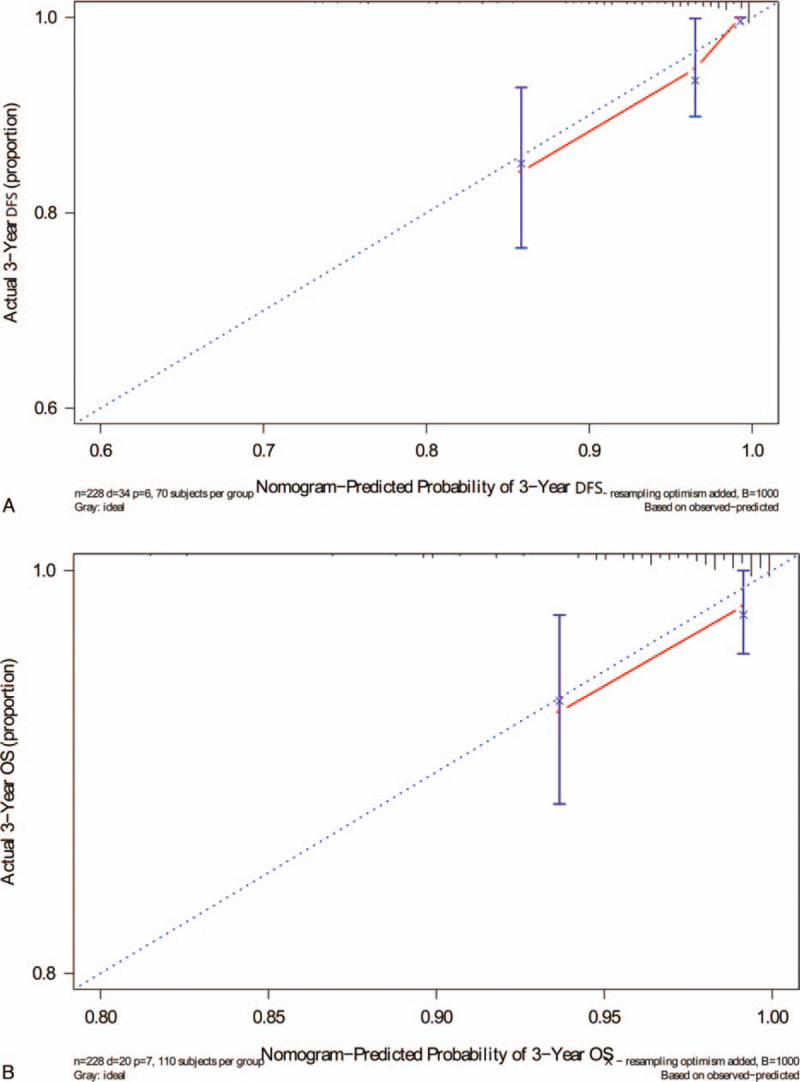
The curves for predicting patient DFS (A) and OS (B) at 3 years. Nomogram model-predicted OS is plotted on the *x*-axis; actual OS is plotted on the *y*-axis. Closer alignment with the diagonal line represents a better estimation. DFS = disease-free survival, OS = overall survival.

To predict DFS and OS for patients with AFR, 2 nomograms were established using the multivariate Cox regression model according to all significantly independent factors for DFS and OS (Fig. 2A and B). Nomograms can be interpreted by summing up the points assigned to each variable, which are indicated at the top of the scale. The total points can be converted to predict 3-, and 5- year DFS and OS for a patient in the lowest scale. C-index of the nomogram based on AFR predicted OS with an accuracy of 0.826, C-index of the nomogram based on AFR predicted DFS with an accuracy of 0.834. The calibration plots were good of illustrating for 3-year DFS and OS (Fig. 3).

### 3.4 The decision curve analysis for DFS and OS

**Figure 4:**
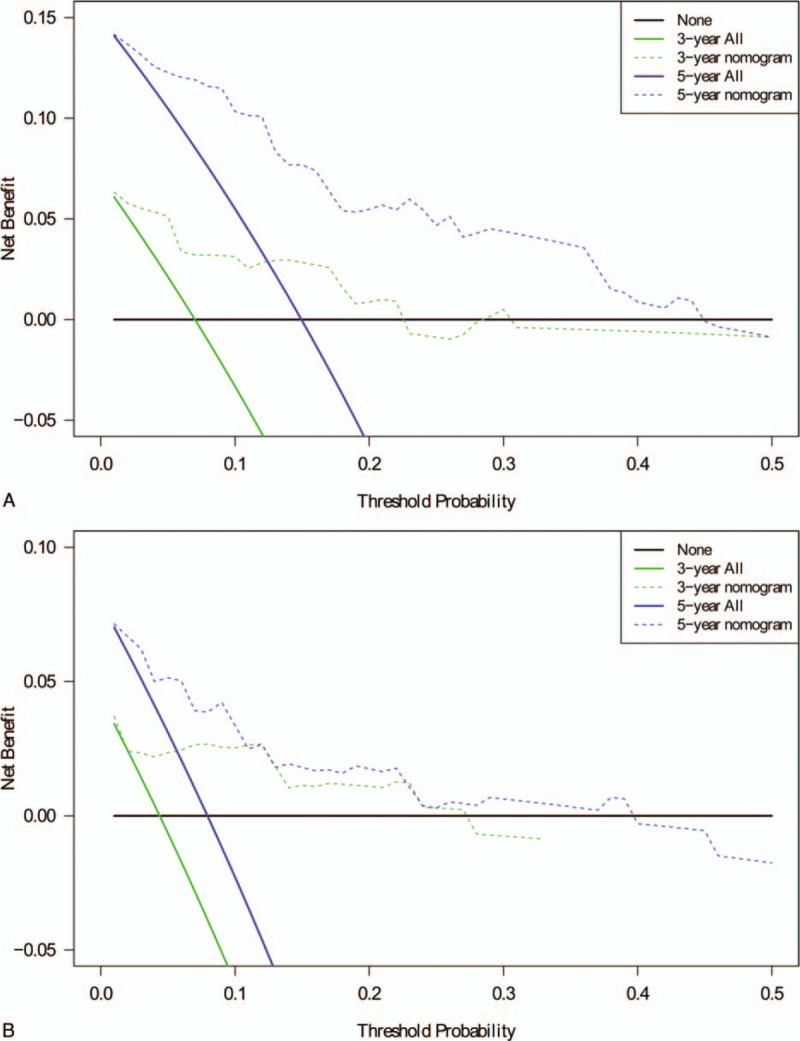
Decision curve analysis for the 2 nomograms in the population, DFS (A) and OS (B). The *y*-axis measures the net benefit. The dotted lines (green and blue) represent the nomogram. The solid lines (green and blue) represent the assumption that all patients have 3-, or 5-year survival and DFS, respectively. The thin black line represents the assumption that no patients have 3-, or 5-year survival and DFS. The net benefit was calculated by subtracting the proportion of all patients who are false positive from the proportion who are true positive. DFS = disease-free survival, OS = overall survival.

In the decision curve analysis, the decision curves were good for predicting the 3- and 5-years of DFS (Fig. 4A) and OS (Fig. 4B).

## 4 Discussion

Fib, which is synthesized by liver, is a crucial component of blood coagulation system via promoting platelet aggregation. Moreover, Fib is reported to be an important biomarker reflecting systemic inflammation.^[14]^ Preoperative low serum Alb is reported to be an indicator for postoperative complications and mortality in patients undergoing transcatheter aortic valve replacement.^[15]^

Several studies have reported that preoperative serum albumin levels were associated with the prognosis of breast cancer. One paper reported that low levels of serum albumin were adversely associated with survival of all stages of breast cancer.^[16]^ Another paper reported that patients with higher albumin level had a 45% reduced risk of death compared with those with lower albumin levels.^[17]^

Few cancer researchers have study AFR. Alb expressions are recommended to be a reliable prognostic factor in patients with cancer.^[2]^ AFR, a ratio of Alb-to-Fib, combines these 2 biomarkers and amplifies the sensitivity for evaluating inflammation and nutrition status. The combination of Alb and Fib is superior to the single Alb and Fib and it has been widely recommended as a prognostic factor in various models, for example, acute ST-segment elevation myocardial infarction (STEMI)^[18]^ and operable soft tissue sarcoma.^[19]^

Our study showed that AFR was a significant and powerful independent prognostic factor in breast cancer. The multivariate Cox regression analysis confirmed the independence of the association between AFR levels and DFS and OS (*P* < .05). In this study, using univariable analysis and subsequent multivariable analysis, we identified WBC, TNM, lymphovascular invasion, nuclear grade, ER/PR(+), P53, and AFR as independent prognostic factors for OS. C-index of the nomogram based on AFR predicted OS with an accuracy of 0.882, and, we identified WBC, TNM, lymphovascular invasion, nuclear grade, P53, Ki-67, and AFR as independent prognostic factors for DFS. C-index of the nomogram based on AFR predicted DFS with an accuracy of 0.922. A value of 0.5 implies that the predictor or model has no discriminatory ability, and a value of 1.0 implies perfect discrimination.^[20]^ A model with an receiver operating characteristic curve of 0.70 to 0.80 is considered good whereas one with an Area Under Curve of 0.80 to 0.90 has excellent discrimination.^[21,22]^ The nomograms performed well with good discrimination and calibration, identifying this model as a simple and easy tool for estimating OS and DFS of individual patients with breast invasive ductal carcinoma.

In this study, we developed 2 nomograms including AFR to improve prognosis prediction for breast invasive ductal carcinoma patients. The nomograms can be used to better predict an individual patient's probability of 3-, and 5-year DFS and OS. The 2 nomograms were performed using calibration plots and the C-index. The nomograms performed well with a good calibration. Furthermore, the C-index for DFS and OS was satisfactory0.922 and 0.884, respectively.

Hwang's et al^[23]^ indicated preoperative fibrinogen to albumin ratio was a strong and significant independent unfavorable prognosticator of breast cancer, especially in stage II/III and luminal A-like subgroups. Our reach was difference from Hwang's et al. The reason maybe is the difference of the s population. Peoperative AFR level is a useful predictor for severe postoperative complications in elderly gastric cancer subjects after radical laparoscopic gastrectomy.^[9]^ This present study was to indicate preoperative AFR as an independent risk factor for DFS and OS in breast invasive ductal carcinoma patients. The close association between inflammation and breast invasive ductal carcinoma might be a possible mechanism.

The present study had several limitations. The sample size was still small. Although we create nomograms, we do not validate them, future studies are needed to externally validate the proposed nomograms.

## 5 Conclusion

The study is to reveal that breast invasive ductal carcinoma patients with high AFR expression had a higher risk of metastasis and poorer survival than patients with low AFR expression. Our results demonstrated that AFR expression in breast invasive ductal carcinoma was an independent predictor of patient outcomes. The 2 nomograms were developed for predicting the probability of 3-, and 5-year DFS and OS. The model might facilitate both clinician and patient counseling and individualized adjuvant treatment decision-making, as well as follow-up scheduling.

## Author contributions

**Conceptualization:** Yunjiang Liu.

**Data curation:** Yaheng Zhao, Yan Yang, Hongsong Zhang.

**Formal analysis:** Feng Liu, Wei Li

**Investigation:** Yaheng Zhao.

**Methodology:** Lihua Zheng, Peng Liu, Feng Liu.

**Visualization:** Feng Liu.

**Writing – original draft:** Lihua Zheng

**Writing – review & editing:** Lihua Zheng, Feng Liu, Yunjiang Liu.
